# Calcium and Vitamin D Supplementation Are Not Associated With Risk of Incident Ischemic Cardiac Events or Death: Findings From the UK Biobank Cohort

**DOI:** 10.1002/jbmr.3375

**Published:** 2018-02-08

**Authors:** Nicholas C Harvey, Stefania D'Angelo, Julien Paccou, Elizabeth M Curtis, Mark Edwards, Zahra Raisi‐Estabragh, Karen Walker‐Bone, Steffen E Petersen, Cyrus Cooper

**Affiliations:** ^1^ MRC Lifecourse Epidemiology Unit University of Southampton Southampton UK; ^2^ NIHR Southampton Biomedical Research Centre University of Southampton and University Hospital Southampton NHS Foundation Trust Southampton UK; ^3^ Université Lille Nord‐de‐France Lille France; ^4^ Portsmouth Hospitals NHS Trust Portsmouth UK; ^5^ NIHR Barts Biomedical Research Centre William Harvey Research Institute Queen Mary University of London London UK; ^6^ NIHR Oxford Biomedical Research Centre University of Oxford Oxford UK

**Keywords:** EPIDEMIOLOGY, ISCHEMIC HEART DISEASE, CARDIOVASCULAR, CALCIUM, VITAMIN D, OSTEOPOROSIS

## Abstract

We investigated associations between calcium/vitamin D supplementation and incident cardiovascular events/deaths in a UK population‐based cohort. UK Biobank is a large prospective cohort comprising 502,637 men and women aged 40 to 69 years at recruitment. Supplementation with calcium/vitamin D was self‐reported, and information on incident hospital admission (ICD‐10) for ischemic heart disease (IHD), myocardial infarction (MI), and subsequent death was obtained from linkage to national registers. Cox proportional hazards models were used to investigate longitudinal relationships between calcium/vitamin D supplementation and hospital admission for men/women, controlling for covariates. A total of 475,255 participants (median age 58 years, 55.8% women) had complete data on calcium/vitamin D supplementation. Of that number, 33,437 participants reported taking calcium supplements; 19,089 vitamin D; and 10,007 both. In crude and adjusted analyses, there were no associations between use of calcium supplements and risk of incident hospital admission with either IHD, or subsequent death. Thus, for example, in unadjusted models, the hazard ratio (HR) for admission with myocardial infarction was 0.97 (95% confidence interval [CI] 0.79–1.20, *p* = 0.79) among women taking calcium supplementation. Corresponding HR for men is 1.16 (95% CI 0.92–1.46, *p* = 0.22). After full adjustment, HR (95% CI) were 0.82 (0.62–1.07), *p* = 0.14 among women and 1.12 (0.85–1.48), *p* = 0.41 among men. Adjusted HR (95% CI) for admission with IHD were 1.05 (0.92–1.19), *p* = 0.50 among women and 0.97 (0.82–1.15), *p* = 0.77 among men. Results were similar for vitamin D and combination supplementation. There were no associations with death, and in women, further adjustment for hormone‐replacement therapy use did not alter the associations. In this very large prospective cohort, there was no evidence that use of calcium/vitamin D supplementation was associated with increased risk of hospital admission or death after ischemic cardiovascular events. © 2018 The Authors. *Journal of Bone and Mineral Research* Published by Wiley Periodicals, Inc.

## Introduction

Calcium supplementation given with or without vitamin D supplementation is widely used, particularly in the elderly, and has been shown to modestly reduce the risk of new fragility fracture,^1^ particularly in those older individuals in residential care.^2^ Calcium and vitamin D supplementation is given routinely as adjunctive therapy with anti‐osteoporosis medications, all of which are licensed to be taken in the context of calcium and vitamin D repletion.^3^ Calcium supplementation either alone or in combination with vitamin D was viewed as extremely safe, other than gastrointestinal side effects and a slightly increased risk of renal stones,^4^ until a publication in the *BMJ* by Bolland and colleagues in 2008. In this New Zealand‐based trial of calcium supplementation in older women, increased risk of myocardial infarction with calcium supplementation was demonstrated.^5^ Subsequent meta‐analyses and a reanalysis of the Women's Health Initiative trial by the same group again demonstrated modest associations between calcium, or calcium and vitamin D, supplementation and increased risk of myocardial infarction but not cardiovascular death.^6^, ^7^ In contrast, no statistically significant associations between calcium and vitamin D supplementation and cardiac outcomes were found in a similar meta‐analysis by Lewis and colleagues^8^ or by investigators studying the Women's Health Initiative.^9^, ^10^, ^11^ However, in the Lewis study, a subanalysis among the much smaller number of individuals randomized to calcium alone did suggest an increased risk of myocardial infarction with use of calcium supplements alone versus placebo. Observational cohort data have led to similarly conflicting findings,^(12–18)^ particularly with regard to dietary calcium intake compared with supplement use. More recently, work has documented differences in the endothelial response to prevailing calcium concentration between healthy and uremic patients,^(19–21)^ and novel Mendelian randomization approaches have indicated an association between lifelong genetically determined calcium concentrations and ischemic heart disease.^22^, ^23^ Given the high incidence of osteoporotic fracture,^24^ cardiovascular side effects of treatments used to improve bone health, such as calcium/vitamin D supplementation, could potentially have major implications for public health.^25^ Across the intervention and observational data, there is substantial heterogeneity among both exposure and outcome definitions, and there is a marked dearth of evidence pertaining to men. We, therefore, undertook a study of sex‐specific associations between calcium and/or vitamin D supplementation and risk of hospitalization for ischemic heart disease, or subsequent mortality in a very large, population‐based cohort of men and women with uniform ascertainment of both exposure and outcomes: the UK Biobank. The large study population also permitted investigation of whether associations might vary according to baseline cardiovascular risk factors.

## Materials and Methods

### Study subjects

We conducted a prospective analysis using data collected in the UK Biobank study, linked to outcome data derived from National Health Service records. Details of the UK Biobank methodology have been published previously.^26^ UK National Health Service (NHS) registers maintain records of almost everybody in the general population (excluding the small number of individuals not legally registered as resident). The protocol is available publicly (http://www.ukbiobank.ac.uk/wp-content/uploads/2011/11/UK-Biobank-Protocol.pdf?phpMyAdmin=trmKQlYdjjnQIgJ%2CfAzikMhEnx6). Using these records, around 9.2 million primary invitations were sent to individuals aged 40 to 69 years living within a reasonable traveling distance of a total of 22 assessment centers across Great Britain in 2007–10.^27^, ^28^ This age range was chosen to allow time for a wide range of incident disease events to accrue, permitting case‐control studies to be undertaken with the aim of investigating the determinants of chronic noncommunicable diseases of middle and later life.

### Data collection

Participants completed a series of touchscreen computer‐based questionnaires followed by a face‐to‐face interview with trained research staff. Details of the assessments and variables are publicly available (http://biobank.ctsu.ox.ac.uk/crystal/), and a transcript of the touchscreen questionnaire may be downloaded (http://www.ukbiobank.ac.uk/wp-content/uploads/2011/06/Touch_screen_questionnaire.pdf?phpMyAdmin=trmKQlYdjjnQIgJ%2CfAzikMhEnx6). The information collected included sociodemographics (age, sex, ethnicity, educational attainment) and lifestyle factors (including cigarette smoking, diet, physical activity, and alcohol use), medication, and supplement use. Physical activity was documented through the question: “In a typical WEEK, how many days did you do 10 minutes or more of vigorous physical activity? (these are activities that make you sweat or breathe hard such as fast cycling, aerobics, heavy lifting).” Height and weight were measured in all participants by trained data collectors during the clinic attendance using standard operating procedures, and body mass index (BMI) was subsequently calculated (kg/m^2^). We obtained information on incident hospital admission coded using the International Statistical Classification of Diseases and Related Health Problems, 10th Revision (ICD‐10) for ischemic heart disease (IHD: I20–I25), myocardial infarction (MI: I21), and death after these events, through linkage to Hospital Episode Statistics and death registry data (Office for National Statistics) with data included up to 10 years after baseline assessment.

This study was conducted under generic approval from the NHS National Research Ethics Service (17th June 2011, Ref 11/NW/0382). Participants provided electronic consent for the baseline assessments.

### Statistical analysis

We performed all analyses in men and women separately. We documented baseline characteristics with the mean (standard deviation) or median (interquartile range) for continuous variables and number (percent) for categorical variables, and testing for differences between those using and not using calcium supplements. We explored associations between calcium and/or vitamin D supplementation and incident hospital admission with ischemic heart disease/myocardial infarction using Cox proportional hazard models, with results expressed as hazard ratios (HRs) and 95% confidence intervals (95% CI). The small proportion of individuals who had reported prior cardiovascular events at baseline, or had linked information on such events before baseline, were excluded because there were insufficient numbers to permit meaningful subanalyses. Results are reported first unadjusted and then adjusted for age, BMI, family history of cardiac disease, smoking, alcohol, educational level, vigorous physical activity, systolic blood pressure, diabetes medications, and cholesterol medications. For women, we also additionally adjusted for use of hormone‐replacement therapy (HRT). Covariates were considered based on known associations with cardiovascular outcomes and included in the models after testing for associations with exposure and outcomes. Analyses were replicated using death from ischemic heart disease or myocardial infarction as outcomes. In a sensitivity analysis, we limited the cohort to those women not taking HRT at baseline.

To further explore whether a healthy user effect might have influenced the findings, we investigated whether there was any interaction between calcium supplementation and either age, BMI, systolic blood pressure, smoking, alcohol, medication for cholesterol, medication for diabetes, medication for hypertension, and dietary calcium intake for the outcome of admission with myocardial infarction.

All analyses were carried out with Stata v 14.2 (StataCorp LP, College Station, TX, USA).

## Results

### Characteristics of the study participants

**Figure 1:**
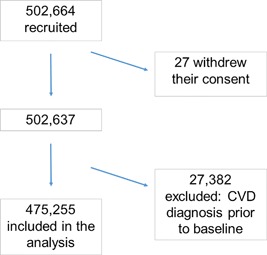
Flowchart of cohort participation.

A total of 502,637 men and women completed the baseline questionnaire and had complete data on calcium and vitamin D intake. Fig. 1 summarizes flow through the cohort from recruitment to analysis. We excluded people who either had hospital admission records for cardiovascular diseases before completing the baseline questionnaire according to the Hospital Episode Statistics or, when recruited, reported to have been diagnosed by a doctor with myocardial infarction or angina. This resulted in a final data set containing 475,255 participants (264,984 women and 210,271 men), all of whom had complete data for the exposure and outcome variables. The average follow‐up time was 7 years and the maximum 10 years. Table 1 shows the characteristics of the study participants at the baseline assessment, and compares those individuals who were taking calcium supplements with those who were not. Overall, the median (IQR) age was 57 (50 to 63) years among women and 57 (50 to 63) years among men, and mean (SD) BMI 27.0 (5.2) and 27.7 (4.2) kg/m^2^, respectively. A total of 59.6% of women and 50.1% of men had never smoked, and 10.6% of women and 2.6% of men took calcium supplements; the use of vitamin D supplements was 5.2% and 2.6%, respectively, with 3.5% women and 0.8% men reporting use of both supplements. Men and women taking calcium supplements as opposed to nonusers tended to be older and are more likely to be taking also vitamin D supplements (Table 1). Medical and lifestyle factors differed very modestly between those taking calcium supplements and those not using them; however, given the very large number of individuals, these differences are statistically significant in most cases. As expected, use of calcium/vitamin D supplementation was positively associated with past history of fracture, with incidence rate ratios (Poisson regression) between 1.2 and 1.8, all *p* < 0.001.

**Table 1:** Characteristics of the Participants

	Women not using calcium supplements	Women using calcium supplements	*p* Value^a^	Men not using calcium supplements	Men using calcium supplements	*p* Value^a^	Cardiovascular risk factor
Demographic characteristics	*n* = 237,020	*n* = 27,964		*n* = 204,798	*n* = 5,473		
Age, years (median [IQR])	57.0 (49.0, 62.0)	60.0 (54.0, 64.0)	<0.001	57.0 (50.0, 63.0)	60.0 (52.0, 65.0)	<0.001	
Body mass index, kg/m^2^ (mean [SD])	27.1 (5.2)	26.0 (4.7)	<0.001	27.7 (4.2)	27.1 (4.2)	<0.001	≥30
Ethnicity (%)							
White	94.7%	92.1%	<0.001	94.3%	89.7%	<0.001	
Qualification (%)							
College or university degree	37.9%	41.6%	<0.001	42.0%	45.2%	<0.001	
A levels/AS levels	14.4%	15.1%		12.6%	12.9%		
O levels/GCSEs	28.5%	26.8%		22.6%	20.1%		
CSEs or equivalent	6.8%	4.5%		6.8%	5.3%		
NVQ or HND or HNC	5.5%	4.5%		10.8%	10.6%		
Other professional qualification	6.9%	7.5%		5.2%	5.9%		
Lifestyle factors							
Alcohol consumption (%)							
At least 3 times per week	37.1%	38.1%	0.001	52.3%	46.4%	<0.001	
Smoking (%)							
Never	59.6%	59.7%	<0.001	50.1%	49.1%	0.007	
Ex	30.7%	33.4%		36.9%	38.9%		
Current	9.1%	6.6%		12.5%	11.4%		✓
Not known	0.6%	0.3%		0.6%	0.7%		
On vitamin D supplements [n (%)]	5358 (2.3)	8373 (29.9)	<0.001	3724 (1.8)	1634 (29.9)	<0.001	
Physical activity, days per week^b^ (median [IQR])	1.0 (0, 3.0)	1.0 (0, 3.0)	<0.001	2.0 (0, 3.0)	2.0 (0, 4.0)	<0.001	
Systolic blood pressure, mmHg (mean [SD])	135.2 (19.2)	135.6 (19.2)	0.005	141.2 917.4)	140.3 (17.9)	<0.001	
Diastolic blood pressure, mmHg (mean [SD])	80.9 (10.0)	80.0 (9.9)	<0.001	84.5 (9.9)	83.5 (10.3)	<0.001	
High blood pressure (>140/90 mmHg) (%)	36.1%	37.2%	<0.001	48.2%	46.6%	0.02	✓
Taking medication for cholesterol (*n* [%])	25387 (10.7)	3308 (11.8)	<0.001	35403 (17.3)	1026 (18.8)	0.005	✓
Taking medication for diabetes (*n* [%])	1712 (0.7)	194 (0.7)	0.59	2368 (1.1)	100 (1.8)	<0.001	✓
Taking medication for hypertension (*n* [%])	38603 (16.3)	4350 (15.6)	0.002	41790 (20.4)	1154 (21.1)	0.22	✓
Family history of cardiovascular events (%)	53.4%	56.1%	<0.001	48.5%	49.7%	0.08	✓
Mean number cardiovascular risk factors	1.5 (1.2)	1.4 (1.1)	<0.001	1.7 (1.3)	1.7 (1.2)	0.12	

^a^ *p* difference in the baseline characteristic between women or men using calcium supplements and women not using supplements; no missing data for these baseline variables. Final column indicates variables included in the count of cardiovascular risk factors.

^b^ In a typical WEEK, how many days did you do 10 minutes or more of vigorous physical activity? (these are activities that make you sweat or breathe hard such as fast cycling, aerobics, heavy lifting).

### Use of calcium, vitamin D, or combined supplementation and incident hospital admission for cardiac events

**Figure 2:**
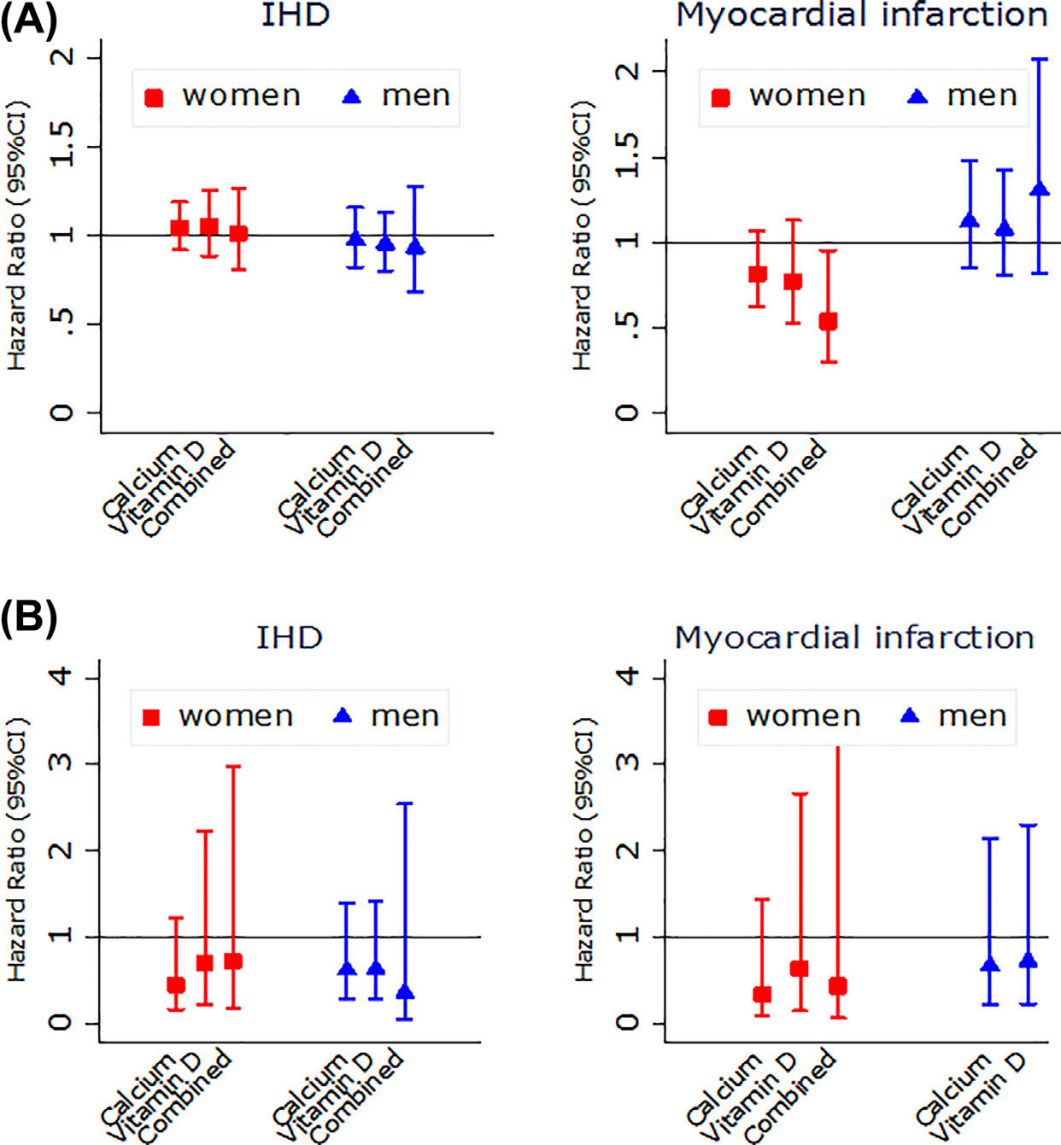
Fully adjusted hazard ratios for (*A*) hospital admission with ischemic heart disease (IHD) or myocardial infarction (MI) among men and women supplemented with calcium, vitamin D, or both, and (*B*) death from ischemic heart disease or myocardial infarction, among men and women supplemented with calcium, vitamin D, or both. The data are the hazard ratio point estimate and 95% CI adjusted for age, BMI, smoking, alcohol, educational level, vigorous physical activity, systolic blood pressure, and diabetes/cholesterol medication.

In total, 7106 men and 3407 women were admitted with ischemic heart disease (IHD); 2456 men and 929 women with myocardial infarction (MI). Table 2 summarizes the hazard ratios for admission with either IHD or MI among men and women separately, associated with use of calcium and/or vitamin D supplementation. The results were broadly similar across all patterns of supplementation. Thus, whether unadjusted or adjusted, the hazard ratio for any outcome, with either of the exposure variables, was not statistically significant. For example, in unadjusted models, the hazard ratio for admission with myocardial infarction was 0.97 (95% CI 0.79–1.20, *p* = 0.79) among women taking calcium supplementation. The corresponding HR for men was 1.16 (95% CI 0.92–1.46, *p* = 0.22). After full adjustment (age, BMI, family history of cardiac disease, smoking, alcohol, educational level, vigorous physical activity, systolic blood pressure, diabetes medications, and cholesterol medications), the HRs (95% CI) were 0.82 (0.62–1.07), *p* = 0.14 among women and 1.12 (0.85–1.48), *p* = 0.41 among men. The adjusted HRs (95% CI) for admission with IHD were 1.05 (0.92–1.19), *p* = 0.50 among women and 0.97 (0.82–1.15), *p* = 0.77 among men. Fig. 2 *A* summarizes these relationships for the fully adjusted data. In women, these null findings remained similar after further adjustment for HRT use; among the 137,750 women who were not using HRT (Table 3), the unadjusted HR (95% CI) for IHD with calcium supplementation was 1.23 (1.05–1.45), *p *= 0.01, but with full adjustment this became 1.18 (0.97–1.43), *p* = 0.11. However, the HR (95% CI) for acute myocardial infarction with calcium supplementation was 1.02 (0.73–1.43), *p* = 0.89, and on full adjustment this became 0.90 (0.60–1.36), *p* = 0.63.

**Table 2:** Associations Between Calcium, Vitamin D, or Combined Supplementation and Incident Hospital Admission for Ischemic Heart Disease or Myocardial Infarction, and Death From Ischemic Heart Disease or Myocardial Infarction

	Women		Men	
	HR (95% CI)	*p* Value	HR (95% CI)	*p* Value
Hospitalization with ischemic heart disease				
*n*	3407		7106	
Calcium				
Unadjusted	1.09 (0.98, 1.21)	0.12	1.05 (0.91, 1.21)	0.48
Fully adjusted	1.05 (0.92, 1.19)	0.50	0.97 (0.82, 1.15)	0.77
Vitamin D				
Unadjusted	1.02 (0.88, 1.18)	0.82	1.02 (0.88, 1.18)	0.82
Fully adjusted	1.05 (0.89, 1.25)	0.56	0.95 (0.80, 1.13)	0.58
Combined				
Unadjusted	0.92 (0.75, 1.12)	0.41	0.86 (0.64, 1.14)	0.30
Fully adjusted	1.01 (0.81, 1.27)	0.93	0.93 (0.68, 1.28)	0.66
Hospitalization with acute myocardial infarction				
*n*	929		2,456	
Calcium				
Unadjusted	0.97 (0.79, 1.20)	0.79	1.16 (0.92, 1.46)	0.22
Fully adjusted	0.82 (0.62, 1.07)	0.14	1.12 (0.85, 1.48)	0.41
Vitamin D				
Unadjusted	0.86 (0.63, 1.17)	0.33	1.08 (0.85, 1.38)	0.53
Fully adjusted	0.77 (0.53, 1.13)	0.18	1.08 (0.81, 1.43)	0.61
Combined				
Unadjusted	0.62 (0.39, 0.99)	0.05	0.96 (0.60, 1.52)	0.85
Fully adjusted	0.54 (0.30, 0.95)	0.03	1.31 (0.82, 2.08)	0.26
Death following ischemic heart disease				
*n*	125		518	
Calcium				
Unadjusted	0.74 (0.39, 1.41)	0.36	1.05 (0.62, 1.78)	0.87
Fully adjusted	0.44 (0.16, 1.22)	0.12	0.62 (0.28, 1.40)	0.25
Vitamin D				
Unadjusted	1.09 (0.51, 2.33)	0.83	0.83 (0.46, 1.51)	0.55
Fully adjusted	0.70 (0.22, 2.23)	0.55	0.63 (0.28, 1.42)	0.27
Combined				
Unadjusted	0.99 (0.37, 2.68)	0.98	0.50 (0.12, 2.00)	0.33
Fully adjusted	0.72 (0.18, 2.97)	0.65	0.36 (0.05, 2.54)	0.30
Death following acute myocardial infarction				
*n*	68		206	
Calcium				
Unadjusted	0.67 (0.27, 1.67)	0.40	1.72 (0.88, 3.36)	0.11
Fully adjusted	0.39 (0.09, 1.65)	0.20	0.78 (0.25, 2.45)	0.67
Vitamin D				
Unadjusted	1.14 (0.42, 3.14)	0.79	0.96 (0.39, 2.32)	0.92
Fully adjusted	0.85 (0.20, 3.56)	0.82	0.79 (0.25, 2.50)	0.69
Combined				
Unadjusted	0.91 (0.22, 3.71)	0.89	0.64 (0.09, 4.58)	0.66
Fully adjusted	0.64 (0.09, 4.69)	0.66	—	—

Fully adjusted models include age, BMI, smoking, family history of cardiac disease, alcohol, educational level, vigorous physical activity, systolic blood pressure, diabetes medications and cholesterol medications.

**Table 3:** Among Women Not Taking Hormone‐Replacement Therapy, Associations Between Calcium, Vitamin D, or Combined Supplementation and Incident Hospital Admission for Ischemic Heart Disease or Myocardial Infarction

	Women	
	HR (95% CI)	*p*
Hospitalization with ischemic heart disease		
*n*	1525	
Calcium		
Unadjusted	1.23 (1.05, 1.45)	0.01
Fully adjusted	1.18 (0.97, 1.43)	0.11
Vitamin D		
Unadjusted	1.02 (0.80, 1.31)	0.86
Fully adjusted	0.99 (0.74, 1.32)	0.94
Combined		
Unadjusted	1.14 (0.84, 1.54)	0.39
Fully adjusted	1.16 (0.82, 1.64)	0.39
Hospitalization with acute myocardial infarction		
*n*	427	
Calcium		
Unadjusted	1.02 (0.73, 1.43)	0.89
Fully adjusted	0.90 (0.60, 1.36)	0.63
Vitamin D		
Unadjusted	0.98 (0.61, 1.57)	0.94
Fully adjusted	0.70 (0.37, 1.32)	0.27
Combined		
Unadjusted	0.92 (0.49, 1.72)	0.78
Fully adjusted	0.69 (0.31, 1.54)	0.36

### Use of calcium, vitamin D, or combined supplementation and death from cardiac events

Table 2 documents risk of death from IHD or MI among men and women using calcium, vitamin D, or both supplements. Consistent with the lack of any statistically significant associations between calcium, vitamin D, or combined supplementation and hospital admission for ischemic cardiovascular events, there was no evidence of any association between these exposures and death from either cause. This lack of association was consistent regardless of adjustment and remained robust in women after inclusion of HRT in the models. Fig. 2 *B* summarizes these associations, using the fully adjusted models.

### Interactions between cardiovascular risk factors and calcium supplementation for admission with myocardial infarction

Tests for interactions between calcium supplementation and baseline characteristics are demonstrated in Table 4 for men and women separately, revealing no statistically significant interactions for the outcome of admission with myocardial infarction.

**Table 4:** The *p* Values for Interactions Between Calcium Supplementation and Baseline Covariates for Admission With Myocardial Infarction in Males and Females (Unadjusted for Other Covariates)

Baseline characteristic	Women	Men
	*p*	*p*
Age (years)	0.41	0.37
Body mass index (kg/m^2^)	0.09	0.15
Smoking (never, ex, current)	0.18	0.18
Dietary calcium intake (mg/d)	0.94	0.43
Alcohol (≥3 times per week)	0.49	0.65
Medication for cholesterol (yes/no)	0.43	0.57
Medication for diabetes (yes/no)	—	0.86
Medication for hypertension (yes/no)	0.37	0.68
Systolic blood pressure (mmHg)	0.92	0.98
Hormone‐replacement therapy use (yes/no)	0.52	—

Shown are *p* values for the test of interaction between calcium supplementation versus no supplementation and baseline covariates for the outcome of admission with myocardial infarction.

## Discussion

We have demonstrated in this large prospective population‐based cohort of women and men that use of calcium and/or vitamin D supplementation was not statistically significantly associated with increased risk of hospital admission or death after ischemic cardiovascular events, specifically after myocardial infarction. This lack of association was robust to adjustment for a range of confounders and similar in both men and women.

Our findings contrast with those of Bolland and colleagues in which an increased risk of myocardial infarction was found in the post hoc analysis of safety reporting in a trial of calcium supplementation in elderly New Zealand women,^5^ through a subsequent meta‐analysis of calcium supplementation trials,^6^ and then calcium and/or vitamin D supplementation trials,^7^ including the Women's Health Initiative (WHI). In the majority of these analyses,^29^ the associations are of borderline statistical significance and inconsistent across vascular outcomes. Indeed, analysis of the WHI by its own investigators, accounting for personal calcium/vitamin D supplementation as did the New Zealand group, did not demonstrate adverse cardiovascular effects.^11^ Consistent with this finding, in the present analysis, we did not observe any interaction between calcium/vitamin D supplementation and background dietary intake. In our analysis, it is notable that the upper bound of the 95% confidence interval for the association between calcium with or without vitamin D and myocardial infarction was 1.07 for women and 1.48 for men (calcium alone), and 0.95 for women and 2.08 for men (combined supplementation). In the Bolland meta‐analysis^30^ including WHI, the relative risk of myocardial infarction ranged from 1.21 to 1.26; given that the vast majority of participants in this meta‐analysis were women, it is apparent that the point estimate is outside the 95% CI for associations among women in our study, although it does fall within the 95% CI for men.

Importantly, the reporting of safety outcomes is heterogeneous between the trials included in the Bolland meta‐analysis and not consistently verified in all.^29^ This is critical because of the well‐documented upper gastrointestinal side effects of calcium supplementation, which may result in misclassification of gastrointestinal as cardiovascular events.^31^ In the present study, outcomes were identified through hospital discharge data and uniformly reported across the whole cohort. Although Bolland and colleagues^32^ reported similar findings when self‐reported outcomes were excluded, no effect of calcium and vitamin D supplementation on cardiovascular outcomes were found by Lewis and colleagues in a further meta‐analysis using only events verified by clinical review, hospital record, or death certificates.^8^ A weak association between calcium supplementation alone and myocardial infarction was observed in a secondary analysis, consistent with the meta‐analyses of Bolland and colleagues. However, this is based on a much smaller number of individuals (*n* = 6333) than the calcium and vitamin D analysis (*n* = 45,796). In the present study, we observed a nonsignificant 18% increase in risk of IHD admission with calcium supplementation amongst women who did not use HRT. Importantly, there was no corresponding trend for myocardial infarction, the outcome most frequently associated with calcium supplementation in previous studies.^29^ Although a recent study suggested associations between calcium supplementation and total cholesterol levels in a relatively small human cross‐sectional study, and that in an ovariectomized rat model a high‐calcium diet increased serum total cholesterol,^14^ to our knowledge there are no human data suggesting that HRT use modifies relationships between calcium supplementation and ischemic heart disease outcomes. Indeed in the WHI, importantly, a trial rather than an observational study, there was no difference in the risk of myocardial infarction/coronary heart disease death by calcium/vitamin D supplementation versus placebo when stratified by use of HRT.^9^ An important consideration in the comparison of these meta‐analyses is that WHI participants were permitted personal use of calcium and vitamin D supplements.^11^ Although Lewis and colleagues did not specifically test for an interaction with personal calcium and vitamin D supplement use in the WHI study, a sensitivity analysis in which the WHI participants using personal supplementation at baseline were excluded yielded very similar null results overall.^8^ This is consistent with the analysis from the WHI investigators^11^ and contrasts with the finding from Bolland and colleagues of a specific effect within the participants who were not taking personal calcium and vitamin D supplementation.^30^

The existing trial data are very largely based on women, and thus the present study complements these findings with a very large cohort of men, who are generally observed to be at higher cardiovascular risk than women of similar ages. Other population‐based cohort studies have derived conflicting findings. The largest existing study including men used the AARP Diet and Health study cohort in which a total of 388,229 men and women aged 50 to 71 years were followed over 12 years.^18^ Here, increasing background intake of dietary calcium was associated with lower risk of death from heart disease. In contrast, supplemental calcium was associated with a 19% increase in heart disease death among men but not women. The US Nurses’ Health Study of 74,245 women followed over 24 years, consistent with our present findings, demonstrated no independent associations between intake of calcium supplements and risk of new coronary heart disease events.^12^ Conversely, in the Heidelberg cohort, participants from the European Prospective Investigation into Cancer and nutrition study (EPIC),^13^ among 23,980 men and women aged 35 to 64 years, there appeared to be an increased risk of myocardial infarction (based on only 7 events), but not cardiovascular death, with the use of calcium‐only supplements but not supplements containing calcium plus other nutrients. A smaller study using the Multi‐Ethnic Study of Atherosclerosis (MESA) cohort of 5448 adults again indicated a decreased risk of incident atherosclerosis with greater dietary calcium intake but a positive association between calcium supplement use and incident coronary artery disease.^17^ A study from Finland demonstrated positive associations between calcium/vitamin D supplementation and coronary heart disease among women but did not consider the baseline intake.^15^ Increased risk of death from all causes and cardiovascular disease but not stroke with increasing calcium intake was observed in a large Swedish cohort.^16^ Conversely, in a large meta‐analysis of calcium/vitamin D trials, this intervention appeared to have a protective effect on mortality.^33^ Given the greater number of individuals in UK Biobank than in these cohorts, we were able to investigate interactions between calcium supplementation and cardiovascular risk factors, in men and women separately. Thus, the lack of association did not appear dependent upon any baseline covariates, and importantly, the population who took calcium supplements seemed broadly similar to nonusers, and if anything might be at slightly higher cardiovascular risk given their greater age, making a “healthy‐user” effect unlikely.

Mechanistic data present a similarly inconclusive picture to those findings from observational cohort and randomized trials. Although several studies have demonstrated associations between serum calcium concentrations and markers of coronary atherosclerosis, the major differences in study design, definition of exposure (eg, calcium, phosphorus, or calcium phosphorus product) and outcome (coronary artery calcification, clinical event), and differences in associations by sex between studies make these data difficult to interpret.^29^ Two recent Mendelian randomization studies have demonstrated positive associations between genetically raised serum calcium concentrations and myocardial infarction.^22^, ^23^ However, the genetic instruments only explained around 0.8% of the variance in calcium concentrations, and these analyses are strongly dependent on the underlying assumptions.^34^ They also represent lifelong exposure to calcium concentration, and this design cannot be used to imply that dietary supplementation in older age or transient increases in calcium concentration lead to these outcomes. Furthermore, it is undocumented, to our knowledge, whether the transient rise in serum calcium concentration after ingestion of a calcium load (which is modest and remains below the saturation point of the calcium × phosphate product) is specifically associated with adverse cardiovascular events.^29^ Given that biological mechanisms sense calcium ions rather than their source, it would seem unlikely that dietary calcium would behave differently to supplements in associations with cardiac outcomes.

Coronary calcification begins with pathological intimal thickening and atherosclerotic plaques forming at sites of endothelial damage.^35^ Calcification of plaques appears to be an active process related to macrophage apoptosis leading to microcalcifications, which may coalesce, rather than dependent upon the prevailing serum calcium concentration.^36^ Although there is evidence from meta‐analyses that calcium‐containing phosphate binders increase ischemic cardiac outcomes in end‐stage renal failure,^20^, ^21^ there is also evidence that the vascular endothelial behaves very differently in this context.^19^ Thus, exposure to raised calcium concentrations leads to calcification of vessels in vascular tissue taken from chronic renal failure patients but not in vascular tissue taken from healthy controls.^19^ Although a recent study demonstrated a potential mechanism of calcium supplementation increasing serum triglycerides in ovariectomized rats,^14^ human studies examining links between calcium supplementation and outcomes such as blood pressure and lipid profile have generally indicated protective relationships.^36^, ^37^, ^38^, ^39^, ^40^

We studied a very large population‐based cohort assessed in detail and with uniform methodology, with outcome events linked through hospital records. However, there are limitations that should be considered in the interpretation of our results. First, calcium and vitamin D supplementation was assessed by self‐report. However, as UK Biobank is not predicated on any individual disease, there is no reason why individuals might preferentially report use of such supplements. Notwithstanding, in the setting of an observational cohort study, residual confounding remains a possibility, and an appropriately powered randomized trial with validated endpoints would ultimately be needed to definitively answer the question. Second, we relied on hospital event linkage for outcome data and therefore may have incomplete capture of nonacute presentations of cardiovascular disease. However, hospital admission for myocardial infarction is a clearly defined and thus more reliable endpoint than its antecedents. Third, we cannot exclude the possibility of selection bias toward a healthy population, as is common with such studies and which is likely to have reduced the incidence of cardiovascular events. Conversely, a healthy population bias is likely to increase use of the exposure, calcium and vitamin D supplementation, and in relation to other studies, this is an extremely large cohort. However, we did not have information on duration of supplement use and therefore could not reliably investigate temporal relationships between the exposure and outcome. Finally, this was not a population of frail elderly individuals, and therefore we cannot exclude associations between supplementation and cardiovascular events in the oldest population.

In conclusion, in this very large prospective UK cohort of around half a million individuals, use of calcium supplementation, with or without vitamin D supplementation, was not associated with increased risk of hospital admission or death after ischaemic cardiovascular events.

## Disclosures

SD'A, JP, EC, ZR‐E, KW‐B, ME, and SP state that they have no conflicts of interes. NH has received consultancy, lecture fees, and honoraria from Alliance for Better Bone Health, AMGEN, MSD, Eli Lilly, Servier, Shire, UCB, Radius, Consilient Healthcare, and Internis Pharma. CC has received consultancy, lecture fees, and honoraria from AMGEN, GSK, Alliance for Better Bone Health, MSD, Eli Lilly, Pfizer, Novartis, Servier, Medtronic, and Roche.
